# Disperse-and-Mix: Oil as an ‘Entrance Door’ of Carbon-Based Fillers to Rubber Composites

**DOI:** 10.3390/nano11113048

**Published:** 2021-11-12

**Authors:** Gal Shachar Michaely, Dimitry Alhazov, Michael Genkin, Matat Buzaglo, Oren Regev

**Affiliations:** 1 Department of Chemical Engineering, Ben-Gurion University of the Negev, Beer-Sheva 84105, Israel; matat.buzi@gmail.com; 2 Alliance Tire Company Ltd., Hadera 38100, Israel; alhazov@gmail.com (D.A.); michagen@gmail.com (M.G.); 3 Ilse Katz Institute for Meso and Nanoscale Science and Technology, Ben-Gurion University of the Negev, Beer-Sheva 84105, Israel

**Keywords:** filler, rubber, composites, mechanical properties, scanning electron microscopy, thermal conductivity

## Abstract

Oil was employed as an ‘entrance door’ for loading rubber with carbon-based fillers of different size and dimensionalities: 1D carbon nanotubes (CNTs), 2D graphene nanoplatelets (GNPs), and 3D graphite. This approach was explored, as a proof of concept, in the preparation of tire tread, where oil is commonly used to reduce the viscosity of the composite mixture. Rubber was loaded with carbon black (CB, always used) and one or more of the above fillers to enhance the thermal and mechanical properties of the composite. The CNT-loaded system showed the best enhancement in mechanical properties, followed by the CNT-GNP one. Rubber loaded with both graphite and GNP showed the best enhancement in thermal conductivity (58%). The overall enhancements in both mechanical and thermal properties of the various systems were analyzed through an overall relative efficiency index in which the total filler concentration in the system is also included. According to this index, the CNT-loaded system is the most efficient one. The oil as an ‘entrance door’ is an easy and effective novel approach for loading fillers that are in the nanoscale and provide high enhancement of properties at low filler concentrations.

## 1. Introduction

Rubbers are viscoelastic polymers in which long-chain molecules are entangled and crosslinked (i.e., vulcanized) to form an elastic material. Rubber is applied in different fields, where more than half of its worldwide production is used in the automotive industry, especially tires [1]. Loading rubber (as a matrix) with particles (termed fillers) of various dimensionalities is used to manipulate the mechanical and thermal properties of the composite [2]. Rubber composites are applied in various applications such as, tires, thermal interface materials, and coating [3,4].

### 1.1. Rubber Loaded with Carbon-Based Fillers

One of the most widely used filler for rubber is carbon black (CB, 3-dimensional structure) [5,6], aiming at enhancing the mechanical properties [2,5,7] (Table 1). There have been various reports on the application of nanocarbons, such as graphene nanoplatelets (GNP, 2D) and carbon nanotubes (CNT, 1D), as fillers for rubbers [8,9,10,11,12,13,14]. These carbon allotropes are characterized with high intrinsic thermal conductivity (TC > 2000 W m^−1^ K^−1^ [15]) and mechanical properties (>300 GPa Young modulus [16] and >10 GPa tensile strength [16], Table 1). Therefore, their loading in rubber is expected to enhance its properties better than CB. Previous reports showed that loading rubber with a single nanocarbon filler in addition to CB yielded enhancement below 30% in TC, compared to CB-rubber composite [8,17,18]. Moreover, rubber systems loaded with more than one filler in addition to CB have not been investigated, although they carry great potential [19,20]. It should be mentioned that in all previously reported studies, the fillers were added directly (as a powder) to the rubber mixture.

A rubber composite consists of rubber, filler, antidegradants, and curative [2], where oil is added in most industrial applications [26,27] to reduce the viscosity of the mixture (i.e., to enhance its processability) [2,26]. Antidegradants (e.g., antioxidants and antiozonant) are added to the rubber to help protect the tire against deterioration by ozone, oxygen, and heat. Curative (sulfur, accelerators, and activators) are used to crosslink the polymer chains (vulcanization) hence transforming the viscous mixture into a strong, elastic material.

### 1.2. Rubber Composites for Tire Tread

#### 1.2.1. The Rubber Matrix

**Figure 1:**
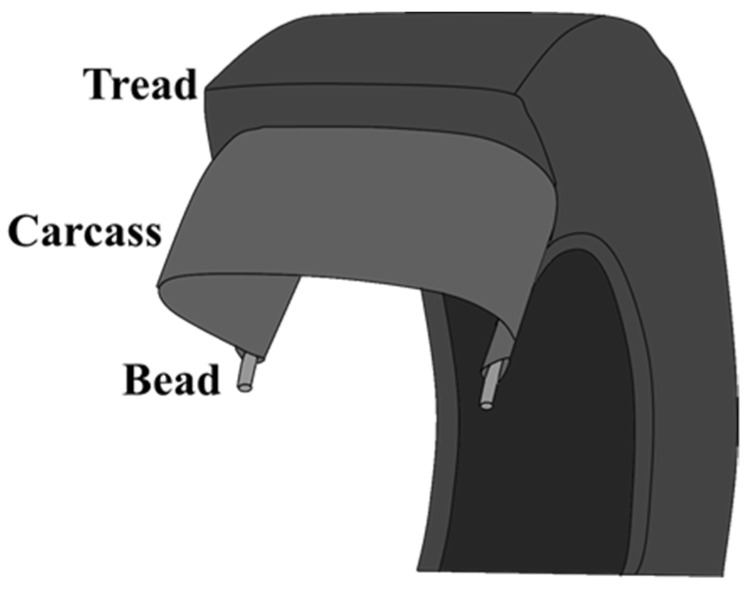
The major components of a tire include bead, carcass, and tread.

A tire consists of bead-in contact with the rim of the wheel, carcass-the framework of the tire, and tread-the focus of this study-is in direct contact with the road and therefore, exposed to extensive abrasion [28] (Figure 1).

The rotation of the tire under the weight of the vehicle results in repeated deformation that generates high friction and consequent elevation in its temperatures [29], e.g., 120 °C was measured in tires of trucks [30]. Therefore, high TC [31] of the tread is essential to efficiently dissipate the heat (vide infra). The tread should resist crack propagation and abrasion resulting in loss of rubber material due to friction with the road [2,32]. It should also have high tensile strength [31] to overcome road conditions that distort its shape.

These properties are interlinked; friction energy is directly converted into heat, resulting in temperature elevation of the tire. The poor thermal conductivity of the tread compound loaded with CB (0.27 W m^−1^ K^−1^) [30] results in a higher rate of heat generation than its dissipation to the ambient air. The temperature increase accelerates the fatigue of the rubber and changes its properties, resulting in significant strength loss, mechanical failures, reduced durability, and abrasion [2]. Therefore, preventing tire overheating is essential, and could be obtained by enhancing its TC and mechanical properties. Fillers such as carbon-based materials are expected to provide the required enhancement in TC [18].

#### 1.2.2. Carbon-Based Fillers

Carbon-based fillers could be categorized by their structure, aspect ratio, and dimensionality [33,34]: GNP-2D and CNT-1D (nano-size fillers, NF). Graphite and CB-3D (micron-size fillers, MF). Except for CB, which consists of a mixture of sp^2^-and sp^3^-hybridized carbon atoms [35], the other fillers mentioned above consist mainly of sp^2^ honeycomb network [35], where the higher the content of sp^2^ bonds, the higher the TC [36]. Therefore, the thermal conductivity of CNT, GNP, or graphite is higher than that of CB (Table 1) in more than two orders of magnitude [15,25]. When compression is applied on a freshly prepared composite, the GNP and graphite are preferentially orientated, hence enhancing the in-plane TC of the composite [36], moreover, higher loading of those filler will resulted in higher TC. Loading a matrix with filler of low dimensionality (usually coupled with higher aspect ratio) results in substantial mechanical reinforcement at lower filler concentration compared to loading of fillers with higher dimensionality [22]. Nevertheless, carbon-based fillers with high dimensionality could serve as an alternative to CB, which is commonly used as filler in the tire industry [37,38,39]. Therefore, the integration of different carbon-based fillers, NF or MF, in rubber could further enhance the performance of the composite at much lower concentrations compared to CB. Our work is aimed at serving as a guide for selection of filler(s) and their dispersion approach in the rubber matrix. We employed the oil as an ‘entrance door’ for various combinations of carbon-based fillers to the rubber mixture, in which oil is the only liquid ingredient at RT. These fillers are efficiently exfoliated and dispersed in oil [40], and therefore, their intrinsic properties are better expressed [34]. We refer to the total filler concentration since we mostly dispersed more than one filler in the oil.

## 2. Materials and Methods

### 2.1. Materials

Natural Rubber grade (SIR-20,PT. Nusa Alam Rubber, Lebak, Indonesia), Low PCA oil (Nytex 4700, NYNAS, Germany), single-wall CNT (1.2–2 nm in diameter, TUBALL MATRIX 603, OCSiAl, Leudelange, Luxembourg), graphite-flakes (Sigma-Aldrich, 33461, Burlington, MA, USA), graphene nanoplatelets (GNP) grade M and H (H15, M25, xG-Sciences, Lansing, MI, USA), carbon black (CB, grade N-220, from OMSK CARBON, Volgograd, Russia), microcrystalline wax (MC Wax H2122B, Shandong Yanggu Huatai Chemical Co. Ltd., Shandong, China), 6PDT (antidegredants) and TBBS (*N*-tert-butyl-benzothiazole sulfonamide), ZnO, and sulfur (curatives) were used as received.

### 2.2. Preparation of the Composites

Dispersions of carbon-based filler in oil were prepared for each filler separately by adding moderately 5–120 g of carbon-based filler to a 50 g oil. The dispersion was mixed in a planetary mixer (Thinky, AR-100, Laguna Hills, CA, USA) with one zirconia ball (10 mm in diameter) for 2 min at 2000 rpm, up to the maximum loading of the filler in the oil above which phase separation was detected (Table 2). The zirconia ball was then removed. The natural rubber, CB, antidegredants, and the filler-oil dispersion were mixed in a laboratory mixer (Buzuluk lab Banbury mixer 2.5 L, 150 °C, 60 rpm, Dalian, Shandong, China). The curatives were then added and mixed for additional 2 min (30 rpm, >100 °C), followed by mixing in a two-roll mill (David Bridge & Co., Ltd., 60 °C, Manchester, UK). The resulting mixture was then cast into variously shaped molds and compressed (300 bar, 143 °C, using rubber compression molding press machine) until vulcanization was completed. The concentrations (by phr) of a reference system (termed REF) was 100:50:10:11.5:2.7 rubber:CB:oil:antidegredants:curative.

### 2.3. Characterization

Measurements of mechanical properties included 100% modulus, tensile strength, and elongation at break. These were measured in Instron 3345 (508 mm/min deformation rate, ASTM D 412-92 [10]). A stress–strain curve for each specimen was obtained from which these properties were calculated.

Abrasion was measured using Gibitre instruments (ASTM D 5936 [41]). The specimen (15 mm in diameter and 8 mm thickness) was inserted into a locking clamp and rotated against a rotating drum to which abrasive paper was attached [41]. The density (Table S1) and the weight were measured before and after the test to calculate the difference in volume, i.e., the abrasion loss (=V_after_ − V_before_). The abrasion resistance is defined as [41]

Abrasion resistance = 1/abrasion loss(1)

TC Measurements were conducted by using a thermal constants analyzer (TPS 500s, Hot Disk, Goteborg, Sweden) based on a transient plane source (TPS) technique [42,43]. In this method, a sensor is placed between two composite samples of the same material. The sensor is heated up while measuring the temperature increase inside the sample over time. The time-dependent change in temperature is used to calculate the TC of the measured material. All measurements are conducted in air at 25 °C [44]. The disc-like specimen size was 44.5 mm in diameter and 12 mm in thickness.

Scanning Electron microscopy (SEM) was carried out using a high-resolution cold-field emission gun SEM (Varios 460L, Oxford EDS, 50 pA, 3 KV, Waltham, MA, USA) operated in secondary electron imaging mode. The sample was cut using a doctor blade, and the exposed surface was examined.

## 3. Results and Discussion

We studied the properties of rubber loaded with combinations of various types of fillers at a wide concentrations range. Then, we compared the different systems based on their integrated enhancements and total filler weight fraction (TFF).

As a proof of concept, we suggested employing the oil as an ‘entrance door’ to the composite for various types of carbon-based fillers (and their combination) to the tread mixture. Our approach was to first disperse the fillers in the oil and then loading the rubber with the stable filler(s)-oil dispersion. They are expected to enhance the performance of the tire by increasing its TC and the mechanical properties such as toughness and tensile strength along with enhanced abrasion resistance.

### 3.1. Rubber Composites Systems

We prepared stock dispersions of the fillers in oil and found higher filler concentration for fillers with higher dimensionality (Table 2). Indeed, the aspect ratio and the dimension of the filler were reported to influence its maximum loading due to viscosity increase [22]; CNTs (1D) tend to entangle (because of their long-wavy structure) and form a network (i.e., increase the viscosity) at lower loading, where GNP (2D) or graphite (3D) are made of stacked platelets that enable them to slip over one another and results only in a moderate increase in viscosity.

Then, we loaded the tread mixture, which already included fixed 50 phr CB, with various combinations of carbon-based materials (Table 3), namely, one, two, or three fillers in the same composite. A stock oil dispersion of each filler was added separately to the tread mixture.

#### Dispersibility of Fillers in the Rubber

**Figure 2:**
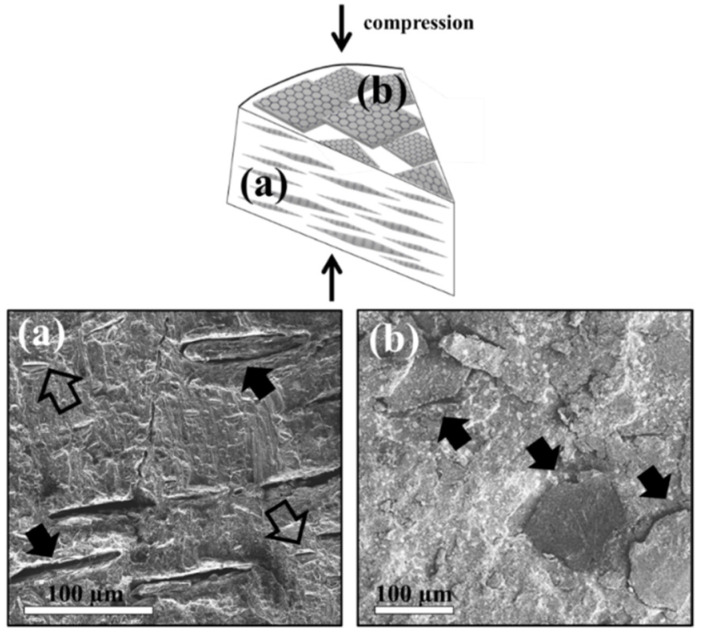
SEM micrographs of rubber loaded with GNP-graphite and CB. (**a**) Cross-section (edge-on): elongated holes indicate pulled-out GNP and graphite (empty and full arrows, respectively). (**b**) Top view (face-on): GNP and Graphite perpendicular to the compression axis (black arrows). The compression vector is indicated in the upper scheme.

SEM imaging indicated that the dispersed GNP and graphite in the rubber composite were not aggregated (Figure 2). The fillers were orientated in the composite (Figure 2) due to the compression process [45] applied during the preparation of the tire (see experimental) [36,45]. At the cross-section (Figure 2a), one can find elongated holes indicating pulled-out GNP or graphite (empty and full arrows, respectively), all in the same orientation. At the top-view (Figure 2b), GNP and graphite are imaged face-on and perpendicular to the compression axis (see schematics).

### 3.2. Thermal Conductivity

**Figure 3:**
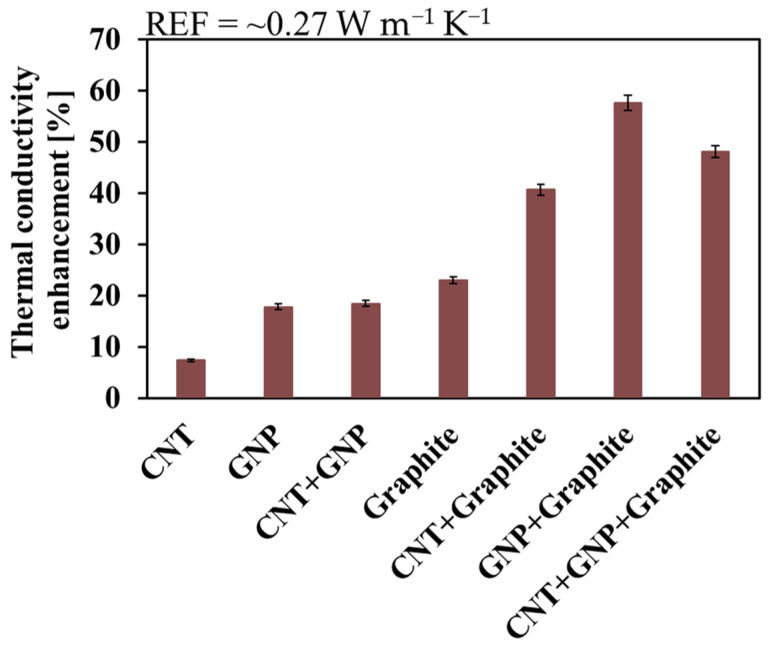
Thermal conductivity enhancement of the various systems. The measured values and their errors are detailed in Table S1.

The TC of the REF tread specimen (loaded with CB only) is rather low (0.27 W m^−1^ K^−1^), thus, enhancing its TC is expected to improve the heat dissipation and reduce its temperature. The various fillers (CNT, GNP, and graphite) and their combinations (Figure 3, abscissa) yielded TC enhancements in the 7–60% range. The CNT system exhibited the lowest TC enhancement, in line with previously reported studies on other matrices [38], followed by GNP. The reason for this is most probably phonon scattering caused by the higher thermal resistance of the CNT over the GNP due to a higher polymer-nanocarbon interface resistance [36,46,47,48,49,50] and higher nanocarbon-nanocarbon contact resistance [36,49].

Higher concentration of graphite (compared to GNP) could be loaded due to its low aspect ratio (Table 1) and the obtained TC was consequently higher. Moreover, the increase in the TC of composites with more than two different fillers (in addition to CB) is probably due to CNT bridging between the graphite or the GNP, or the reduced contact resistance between GNP and graphite, especially after compression [45].

### 3.3. Mechanical Properties

Since the tire rotates underweight and repeatedly deforms and recovers, high elasticity (modulus) and tensile strength [2] are essential for endurance in rough road conditions (300 psi and 3700 psi, respectively). Moreover, high values of elongation at the break of the tire implies that the tire could tolerate severe deformations without failure [2].

**Figure 4:**
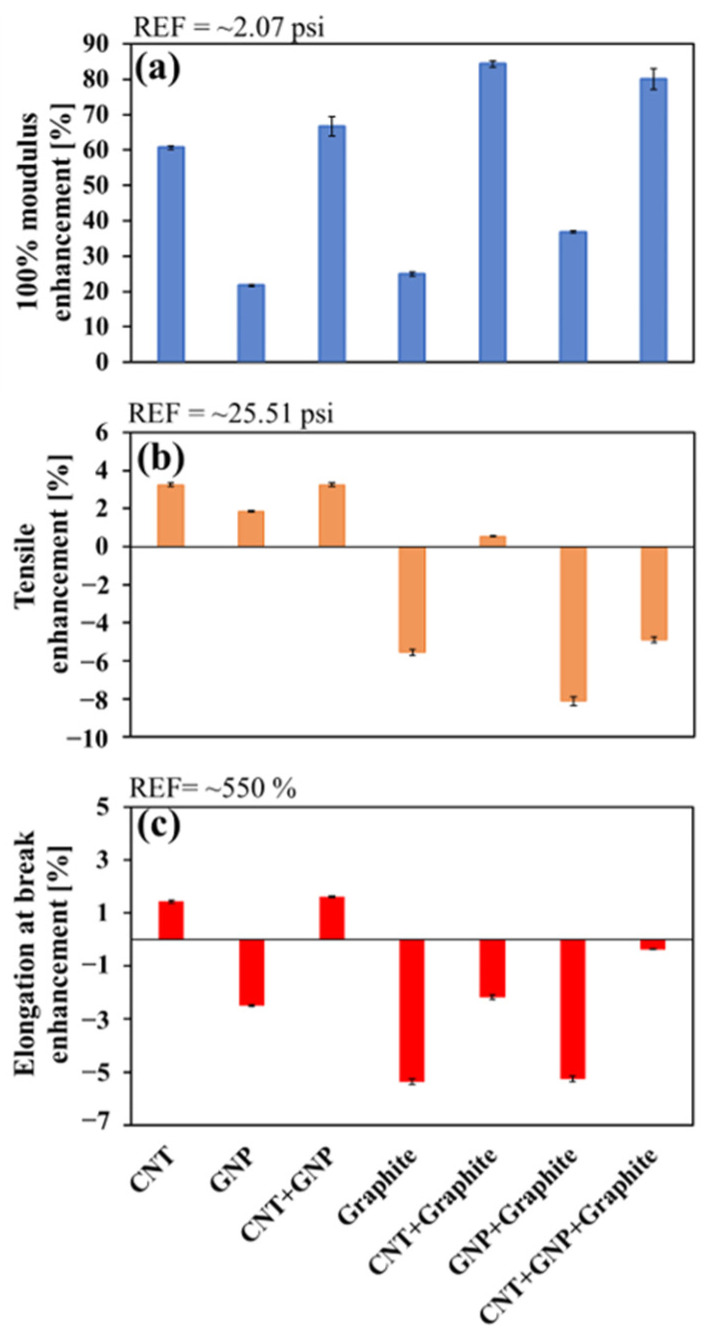
Mechanical properties enhancement of the various systems: (**a**) 100% modulus (blue), (**b**) tensile strength (orange), and (**c**) elongation at break (red). The measured values and their errors are detailed in Table S1.

There are conflicting reports on the effect of loading carbon-based fillers on the mechanical properties of rubber-based composites. Some papers reported only on enhanced modulus but reduced elongation at break and tensile strength [16,51], while others indicated enhancement in all properties [52,53,54]. Therefore, we systematically studied a wide spectrum of mechanical properties (Figure 4) to provide a common ground for comparing the different combinations of composite systems to enhance the overall tread properties.

The CNT system showed an enhancement in all forms of mechanical properties (Figure 4), while the GNP system showed a slight decrease in the elongation at break and minor enhancement in the other mechanical properties. This provides a significant mechanical advantage of the CNT over the GNP-loaded systems, due to the CNT ability to form a network structure [22].

The presence of CNT is essential since in its absence (e.g., GNP-graphite system) most mechanical performances (besides 100% modulus) are degraded. All graphite-containing systems do not show substantial enhancements in the mechanical properties of the tread. Thus, loading of 3D filler (Graphite) does not offer any enhancement in the mechanical performance at this range of concentration (<5.38 × 10^−2^ weight fraction of graphite) dictated by the low concentration of oil (see Section 2.2, Table 3).

In the CNT-GNP system all mechanical properties are also enhanced due to synergistic effect of the 1D and 2D fillers (CNT-GNP hybrid), in keeping with previously reported epoxy-based nanocomposite, where it was suggested that CNTs bridged between neighboring GNPs [19,47].

In summary, CNTs (1D) reinforced the matrix at very low concentrations (5.4 × 10^−3^ weight fraction) due to their high aspect ratio and the formation of strong filler-filler network [53]. In that respect, fillers with higher dimensionalities (e.g., 2D and 3D) achieve the maximal mechanical enhancement at higher fillers loading [22].

### 3.4. Abrasion Resistance

**Figure 5:**
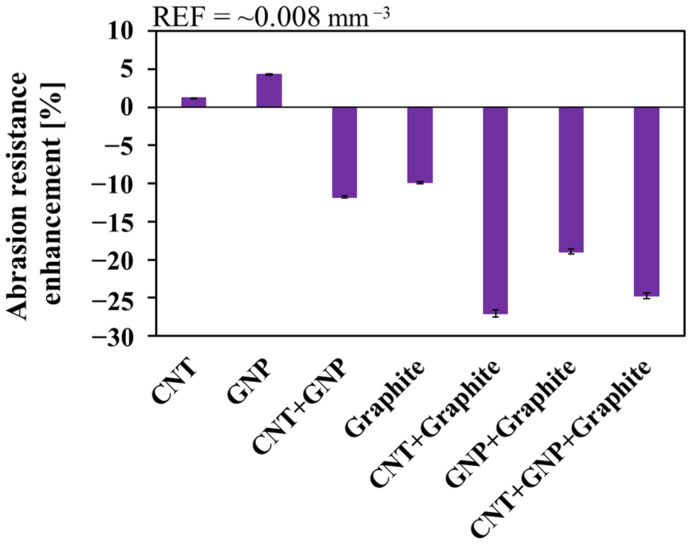
Abrasion resistance enhancement of the different systems. The measured values and their errors are detailed in Table S1.

Abrasion resistance directly relates to the service life of the tire. It is defined as the ability of a surface to resist rubbing or friction [55] and should be as high as possible to achieve better mileage and durability [29]. Here, both CNT and GNP systems demonstrated minor enhancement (Figure 5), where all the other systems showed massive degradation (>10%).

### 3.5. The Overall Efficiency of the Various Systems

An efficient system is expected to enhance all mechanical and thermal properties at low filler concentration to achieve maximum enhancement with minimum loading of filler(s). We, therefore, suggest an overall relative efficiency (ORE, dimensionless) in which all relative properties (X_r_ = X_composite_/X_REF_) are integrated and divided by the TFF (Table 3):

ORE = ∑X_r_/TFF (2)

X_r_ > 1 and X_r_ < 1 indicate enhancement and degradation of a property, respectively. Therefore, properties that were degraded are given negative values.

**Figure 6:**
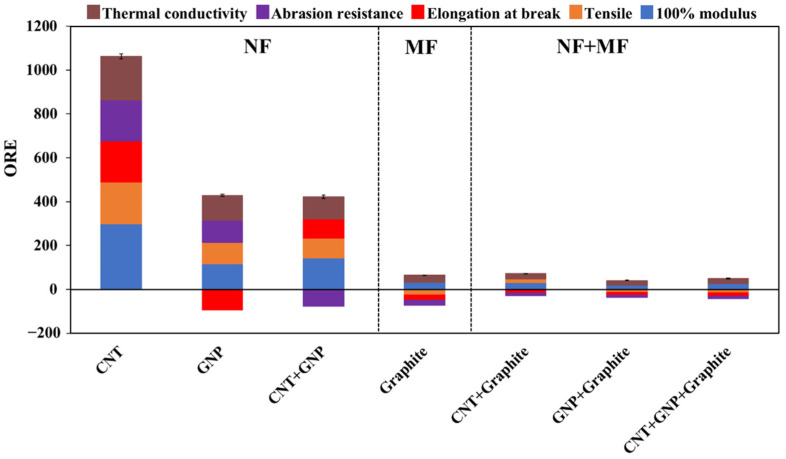
The overall R\relative E\efficiency (ORE) values of the various studied combination of fillers (see composition in Table 3). The color code indicates the contribution of the different properties (as in Figure 3, Figure 4 and Figure 5). The dashed lines separate the nano-size filler (NF), micron-size filler (MF), and their combination (NF + MF).

In that way, we can compare the ORE indices of systems loaded with nanometer size filler (NF), micron-size filler (MF), and their combination. It is clearly shown (Figure 6) that the CNT-loaded composite yielded the highest ORE value (1060, at 5.4 × 10^−3^ weight fraction of CNT, Table 3), and it is the only system that enhanced all the examined properties, followed by the CNT-GNP and GNP systems that did not enhance the abrasion resistance and elongation at break, respectively. All the other systems, including graphite (MF), have roughly the same low ORE values, where most properties were degraded. As mentioned, this could be due to the highest aspect ratio of CNT (Table 1) and NF in general that resulted in high enhancement at low filler loading [53], in agreement with previous reports for other matrices [16,22].

## 4. Conclusions

A dispersion of exfoliated carbon-based fillers in oil was employed as an ‘entrance door’ to a typical tread mixture in the tire industry. Loading a tread mixture with relatively low CNT concentration (5.4 × 10^−3^ weight fraction) substantially enhanced all examined mechanical properties by 2–60% (Figure 4), followed by CNT-GNP and GNP systems that did not enhance abrasion resistance and elongation at break, respectively. Furthermore, the presence of graphite, MF, in the system decreased the mechanical performance of the composite at higher loadings (up to 5.38 × 10^−2^ weight fraction of graphite). Therefore, only the NF systems have shown enhanced performance.

The TC values of the various composites (Figure 3) were mostly affected by the TFF of the fillers and their compression process-derived orientation (Figure 2). The isotropic structure of the graphite enabled one to load much higher TFF without reducing the processability (increased viscosity). Still, the achieved TC values (Table S1) are rather low since the maximum concentrations of the carbon-based filler in the composite were limited by their maximal concentration in the oil (Table 2), the low oil concentration in the tread mixture (10 phr) and, consequently, the low filler concentration in the composite (Table 3).

A CNT-GNP rubber-based system showed superior mechanical performance, in agreement with reports on epoxy-based composites [52,56,57]. In that respect, CNT system has a better abrasion resistance than CNT-GNP system (Figure 5). Moreover, comparing the different systems by the ORE index (Equation (2)) indicated that the CNT system has the best overall performance, followed by CNT-GNP and GNP systems. The ORE values of NF-loaded systems are substantially higher than those of the MF and NF + MF systems (Figure 6).

The oil as an ‘entrance door’ of the fillers to the rubber-based composites is a rather facile, convenient, and fully applicable approach. Therefore, it should preferably be applied to fillers with a high ORE value (i.e., high enhancement at low filler loading), such as CNT. The obtained ORE values (Figure 6) indicate that the discussed approach is mostly applicable to fillers in the nanoscale (NF).

## Figures and Tables

**Table 1:** Properties of carbon-based fillers: carbon nanotube (CNT), graphene nanoplatelets (GNP), graphite, and carbon black (CB).

	CNT	GNP	Graphite	CB
Dimensionality	1D	2D	3D	3D
Structure	cylindrical	Plate-like	Stacked plates	Ball-like
Aspect ratio	2000 [21]	320 [22]	1 [22]	~1 [23]
Intrinsic Thermal conductivity [W m^−1^ K^−1^]	3000 [24]	2000 [24]	2000 [24]	1 [25]
Intrinsic Tensile strength [GPa]	15–150 [16]	20–200 [16]		

**Table 2:** Maximum loading of the different fillers in the oil dispersion.

	CNT	GNP	Graphite
Maximum loading in oil [weight fraction]	0.10	0.28	0.77

**Table 3:** The concentration (in weight fraction) of the carbon-based fillers in the tread rubber mixture and the total filler weight fraction (TFF). All systems include 50 phr CB.

	REF	CNT	GNP	CNT + GNP	Graphite	CNT + Graphite	GNP + Graphite	CNT + GNP + Graphite
CNT	-	5.4 × 10^−3^	-	5.4 × 10^−3^	-	5.0 × 10^−3^	-	4.9 × 10^−3^
GNP	-	-	1.04 × 10^−2^	6.2 × 10^−3^ (H-15)	-	-	1.13 × 10^−2^ (H-15)	6.8 × 10^−3^ (H-15)
Graphite	-	-	-	-	3.86 × 10^−2^	5.38 × 10^−2^	6.13 × 10^−2^	5.38 × 10^−2^
TFF	0	5.4 × 10^−3^	1.04 × 10^−2^	1.16 × 10^−2^	3.86 × 10^−2^	5.88 × 10^−2^	7.26 × 10^−2^	6.55 × 10^−2^
		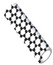	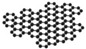	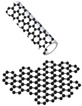	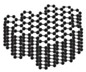	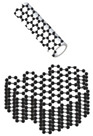	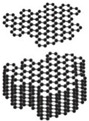	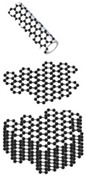

## Data Availability

The data presented in this study are available in Supplementary Materials.

## Supplementary Materials

The following are available online at https://www.mdpi.com/article/10.3390/nano11113048/s1, Table S1: Measured values of the examined systems. A reference sample was prepared and measured for each system (REFx).
