# Supplementary material for: Disperse-and-Mix: Oil as an ‘Entrance Door’ of Carbon-Based Fillers to Rubber Composites
Source: Nanomaterials (Basel). 2021 Nov 12;11(11):3048. doi: 10.3390/nano11113048 (PMC8619991; doi:10.3390/nano11113048)
Supplement: Supplementary file 1 [file nanomaterials-11-03048-s001.zip › nanomaterials-1437341-supplementary.pdf]

Supplementary Materials

Disperse-and-Mix: Oil as an 'Entrance Door' of Carbon-Based Fillers to Rubber Composites

Gal Shachar Michaely [1,*], Dimitry Alhazov [2], Michael Genkin [2], Matat Buzaglo [1] and Oren Regev [1,3,*]

[1] Department of Chemical Engineering, Ben-Gurion University of the Negev, Beer-Sheva 84105, Israel; matat.buzi@gmail.com
[2] Alliance Tire Company Ltd., Hadera 38100, Israel; alhazov@gmail.com (D.A.); michagen@gmail.com (M.G.)
[3] Ilse Katz Institute for Meso and Nanoscale Science and Technology, Ben-Gurion University of the Negev, Beer-Sheva 84105, Israel
* Correspondence: galsh306@gmail.com (G.S.M.); oregev@bgu.ac.il (O.R.)

- **Table S1.** Measured values of the examined systems. A reference sample was prepared and measured for each system (REF$_x$).

	**100% modulus**		**Tensile strength**		**Elongation at break**		**Abrasion resistance**		**Thermal conductivity**		**Density [g cm^{-3}]**
	**Measured value [MPa]**	**Enhancement [%]**	**Measured value [MPa]**	**Enhancement [%]**	**Measured value [%]**	**Enhancement [%]**	**Measured value [mm^{-3}]**	**Enhancement [%]**	**Measured value [W m^{-1}K^{-1}]**	**Enhancement [%]**	
**REF$_{CNT}$**	2.07 ± 0.07	-	25.4 ± 0.6	-	552 ± 7	-	0.0085 ± 0.0001	-	0.27 ± 0.01	-	1.093 ± 0.001
**CNT**	3.32 ± 0.03	60.7 ± 0.5	26.2 ± 0.7	3.26 ± 0.09	560 ± 20	1.45 ± 0.05	0.0086 ± 0.0001	1.18 ± 0.01	0.29 ± 0.01	7.4 ± 0.2	1.095 ± 0.001
**REF$_{GNP}$**	2.10 ± 0.03	-	26.2 ± 0.7	-	560 ± 10	-	0.0070 ± 0.0001	-	0.28 ± 0.01	-	1.093 ± 0.001
**GNP**	2.55 ± 0.04	21.7 ± 0.4	26.7 ± 0.4	1.84 ± 0.02	546 ± 4	−2.50 ± 0.02	0.0073 ± 0.0001	4.29 ± 0.06	0.33 ± 0.01	17.9 ± 0.5	1.096 ± 0.001
**REF$_{CNT+GNP}$**	2.07 ± 0.07	-	25.4 ± 0.6	-	552 ± 7	-	0.0085 ± 0.0001	-	0.27 ± 0.01	-	1.093 ± 0.001
**CNT+ GNP**	3.4 ± 0.1	67 ± 2	26.2 ± 0.7	3.26 ± 0.08	561 ± 7	1.63 ± 0.02	0.0075 ± 0.0001	−11.8 ± 0.2	0.32 ± 0.01	18.5 ± 0.6	1.096 ± 0.001

**REF$_{Graphite}$**	2.21 ± 0.07	-	24.8 ± 0.7	-	560 ± 10	-	0.0081 ± 0.0001	-	0.26 ± 0.01	-	1.093 ± 0.001
**Graphite**	2.76 ± 0.07	25.0 ± 0.6	23.4 ± 0.7	−5.6 ± 0.2	530 ± 10	−5.4 ± 0.1	0.0073 ± 0.0001	−9.9 ± 0.1	0.32 ± 0.01	23.1 ± 0.7	1.105 ± 0.001
**REF$_{CNT+Graphite}$**	2.07 ± 0.07	-	25.4 ± 0.6	-	552 ± 7	-	0.0085 ± 0.0001	-	0.27 ± 0.01	-	1.093 ± 0.001
**CNT+ Graphite**	3.67 ± 0.04	84.3 ± 0.9	25.5 ± 0.7	0.54 ± 0.01	540 ± 20	−2.17 ± 0.08	0.0062 ± 0.0001	−27.1 ± 0.4	0.38 ± 0.01	41 ± 1	1.111 ± 0.001
**REF$_{GNP+Graphite}$**	1.99 ± 0.06	-	25.5 ± 0.7	-	570 ± 20	-	0.0074 ± 0.0001	-	0.26 ± 0.01	-	1.093 ± 0.001
**GNP+ Graphite**	2.72 ± 0.02	36.8 ± 0.3	23.4 ± 0.7	−8.1 ± 0.2	540 ± 10	−5.3 ± 0.1	0.0060 ± 0.0001	−18.9 ± 0.3	0.41 ± 0.01	58 ± 1	1.112 ± 0.001
**REF$_{CNT+GNP+Graphite}$**	2.07 ± 0.07	-	25.4 ± 0.6	-	552 ± 7	-	0.0085 ± 0.0001	-	0.27 ± 0.01	-	1.093 ± 0.001
**CNT+GNP+ Graphite**	3.7 ± 0.1	80 ± 3	24.1 ± 0.7	−4.9 ± 0.1	550 ± 20	−0.36 ± 0.01	0.0065 ± 0.0001	−24.7 ± 0.4	0.4 ± 0.01	48 ± 1	1.113 ± 0.001
